# A Multilayered Imaging and Microfluidics Approach for Evaluating the Effect of Fibrinolysis in *Staphylococcus aureus* Biofilm Formation

**DOI:** 10.3390/pathogens12091141

**Published:** 2023-09-06

**Authors:** Raffaella Parente, Maria Rita Fumagalli, Alessia Di Claudio, Cindy Lorena Cárdenas Rincón, Marco Erreni, Damiano Zanini, Giacomo Iapichino, Alessandro Protti, Cecilia Garlanda, Roberto Rusconi, Andrea Doni

**Affiliations:** 1 Multiscale ImmunoImaging Unit (mIIu), IRCCS Humanitas Research Hospital, Via Manzoni 56, 20089 Rozzano, Italy; 2 Department of Biomedical Sciences, Humanitas University, Via Rita Levi Montalcini 4, 20090 Pieve Emanuele, Italy; 3 Department of Anesthesia and Intensive Care Units, IRCCS Humanitas Research Hospital, Via Manzoni 56, 20089 Rozzano, Italy; 4 IRCCS Humanitas Research Hospital, Via Manzoni 56, 20089 Rozzano, Italy

**Keywords:** *Staphylococcus aureus*, biofilm, innate immunity, extracellular matrix, hemostasis, fibrinolysis

## Abstract

The recognition of microbe and extracellular matrix (ECM) is a recurring theme in the humoral innate immune system. Fluid-phase molecules of innate immunity share regulatory roles in ECM. On the other hand, ECM elements have immunological functions. Innate immunity is evolutionary and functionally connected to hemostasis. *Staphylococcus aureus* (*S. aureus*) is a major cause of hospital-associated bloodstream infections and the most common cause of several life-threatening conditions such as endocarditis and sepsis through its ability to manipulate hemostasis. Biofilm-related infection and sepsis represent a medical need due to the lack of treatments and the high resistance to antibiotics. We designed a method combining imaging and microfluidics to dissect the role of elements of the ECM and hemostasis in triggering *S. aureus* biofilm by highlighting an essential role of fibrinogen (FG) in adhesion and formation. Furthermore, we ascertained an important role of the fluid-phase activation of fibrinolysis in inhibiting biofilm of *S. aureus* and facilitating an antibody-mediated response aimed at pathogen killing. The results define FG as an essential element of hemostasis in the *S. aureus* biofilm formation and a role of fibrinolysis in its inhibition, while promoting an antibody-mediated response. Understanding host molecular mechanisms influencing biofilm formation and degradation is instrumental for the development of new combined therapeutic approaches to prevent the risk of *S. aureus* biofilm-associated diseases.

## 1. Introduction

An interplay between hemostasis and inflammation is essential in the host defense against pathogens [1,2]. The activation of coagulation and fibrinolysis occurs during both acute and chronic bacterial infections [3,4,5]. Coagulation and fibrin formation exert direct antimicrobial functions by physically entrapping bacteria or encapsulating bacterial foci within infected tissue, thus limiting dissemination [6], or by regulating the local inflammatory response [7]. Components of the humoral innate immunity system affect the hemostatic response [8,9]. The inflammation-induced activation of coagulation pathways is initially beneficial, even contributing to antimicrobial defense [10], but when deregulated, coagulation may lead to widespread microvascular thrombosis and tissue damage [11].

The involvement of the extracellular matrix (ECM) elements in the innate immune response is a recurring theme. Indeed, although ECM and coagulation molecules are not considered part of innate immunity, the evasion of pathogens from host defense includes mechanisms mediated by their interaction with the ECM, as well as hemostasis [12,13,14]. On the other hand, elements of the ECM display immunological functions, such as acting as opsonins for certain microbial species [12,14,15]. This suggests a mutually dependent functionality between the ECM and innate immune system [16].

*Staphylococcus aureus* (*S. aureus*) is a pervasive Gram-positive bacterium, a common cause of bacteremia and responsible for several diseases, with a case-fatality rate of 20–25% [17]. *S. aureus*-related infections range from minor skin infections to serious, life-threatening conditions, such as endocarditis, pneumonia and sepsis [18]. The emergence of antibiotic-resistant strains of *S. aureus*, such as methicillin-resistant *S. aureus* and vancomycin-resistant *S. aureus*, has renewed the interest in better defining mechanisms of pathogen virulence and host defense [19,20]. The pathogenic potential of *S. aureus* includes immune evasion strategies based on the interaction with elements of the ECM and hemostasis, through the expression of a variety of surface proteins and specific proteases. Different *S. aureus* virulence factors specifically affect the host’ hemostasis [21,22]. In blood, *S. aureus* coagulases are essential to forming a mechanical barrier to protect *S. aureus* from recognition by opsonins and phagocytes [23,24] and act as crucial determinants for dissemination [22,25]. In tissue, *S. aureus* staphylokinase interacting with plasminogen (PLG) plays a key role in dissemination, causing multi-organ dysfunction syndrome [26]. Immunization against these molecules protects against disease in mice [27]. Moreover, *S. aureus* interacts with elements of the ECM, such as fibronectin (FN), which allows for invasion into different cell types via the α5β1 integrin [13].

A biofilm refers to a community of bacteria in which surface-exposed proteins, called microbial surface components recognizing adhesive matrix molecules, initiate attachment to biotic or abiotic surfaces [28,29,30]. Biofilm is composed of a self-secreting matrix of extracellular polymeric substances, including polysaccharides and extracellular DNA (eDNA), forming an ECM that encloses bacteria and anchors them to the surface of an implant [31]. The interaction of *S. aureus* with host molecules present in the blood and ECM also influences biofilm formation by promoting adhesion and aggregation [32]. In particular, several studies have shown that in situ fibrin formation is a constituent of the biofilm matrix, and *S. aureus*-induced coagulation through the action of coagulase is important in the initiation stages of the process and in biofilm establishment [28,33]. *S. aureus* coagulase-mediated biofilm exhibits increased resistance to immune recognition and antimicrobial treatment [34].

*S. aureus* biofilm-related infections represent a medical need, given the extensive use of indwelling medical devices (such as prosthetic heart valves, orthopedic implants and intravascular catheters) in modern medicine [35]. Microorganisms that grow attached to the surface of an implant or medical device are estimated to be responsible for 60–70% of all hospital-acquired infections, and most of them are related to *S. aureus* or *Staphylococcus epidermidis* [28]. Biofilms can negatively interfere with device function, damage surrounding tissues, cause inflammation and eventually colonize adjacent body sites [29]. Infections related to biofilms are particularly difficult to treat due to their structure, allowing them to evade the immune response and favoring antibiotic resistance. Protocols for preventing biofilm formation, including the use of antibacterial coatings and nanostructured materials, have been applied [28,29], but the development of new approaches to the prevention, treatment and management of biofilm-related infections remains crucial.

*S. aureus*-driven molecular mechanisms underlying processes leading to biofilm formation have been extensively investigated [31]. However, the role of the engagement of host molecules by *S. aureus* is not exhaustively described. Evidence points to a functional relationship between ECM and hemostasis in the initiation of biofilm [32]. The addition of fibrinogen (FG) to coagulase-positive *S. aureus* cultures promotes biofilm formation acting on early stages of adhesion and clotting [36].

Microfluidic devices have emerged as a powerful tool for mimicking in vivo hydrodynamic conditions in biofilm-related studies [37]. They enable long-term assays and real-time dynamic analyses [38] and offer more precise control over relevant parameters (such as fluid flow and surface properties), as close as possible to actual clinical conditions in patients. Moreover, the use of geometrical confinement in microfluidic channels has yielded valuable insights into the behavior of microbes at the single-cell level [39]. Studies based on different microfluidics tools are essential in deepening our understanding of biofilm formation and potential strategies for their management [40].

The present study is aimed at expanding the knowledge of the underlying mechanisms that lead to *S. aureus* biofilm formation by dissecting the complex process. For the purpose, we combined Live Cell Imaging, microfluidics and data analysis in order to investigate the role of elements of the ECM and hemostasis in the different phases leading to *S. aureus* biofilm formation. Using different microfluidics approaches, we defined FG as an essential molecule in the *S. aureus* activities during adhesion, coagulation and matrix assembly, biofilm formation and constitution. We also described a role of fibrinolysis in interfering with biofilm formation and in promoting immunoglobulin (IgG)-mediated immune responses that lead to pathogen killing in plasma from septicemic patients. The results provide a better understanding of the mechanisms at the basis of *S. aureus* biofilm formation, mediated by the interaction between *S. aureus* and host molecules and instrumental in the development of new combined therapeutic strategies for preventing *S. aureus* biofilm-associated infections and sepsis.

## 2. Materials and Methods

### 2.1. Ethics Statement and Clinical Samples

Acid-citrate-dextrose (ACD)-plasma of patients was collected after a positive bacteriological diagnosis for *S. aureus* infection by the clinical personnel of the Intensive Care Unit (ICU) in Humanitas Research Hospital under Ethic Statement Approval n° 820/18. One patient with septic shock from osteomyelitis undergoing multiple surgeries complicated by infective endocarditis of the mitral valve (Pz 1) and a patient with bacteremia and sepsis from an epidural abscess (Pz 2) were included for the sample collection. Informed consent was obtained from all subjects involved in the study. The levels of FG in the ACD-plasma vs. serum of normal donors (n = 3) were measured by ACL TOP^®^ 750 CTS (Werfen, Milan, Italy).

### 2.2. Staphylococcus aureus

*S. aureus* Newman strain tagged with green fluorescent protein (GFP) was gently sourced by Prof. S. Foster [41] (Florey Institute for Host Pathogen Interactions; University of Sheffield, School of Biosciences). *S. aureus* was grown in Tryptic Soy Broth (TSB, Tryptone Soya Broth, CASO Broth, Soybean Casein digest Broth, Casein Soya Broth; STBMTSB12 Millipore, Darmstadt, Germany) under the resistance of kanamycin (10 µg/mL; Sigma-Aldrich, Darmstadt, Germany) at 37 °C and 200 rpm and collected on the day of the experiment at an optical density (O.D.) of 0.6 at A_600nm_ (1 × 10^8^ CFU/mL), measured using a GeneQuant spectrophotometer (Amersham Biosciences, Little Chalfont, UK). Immediately before performing the microfluidics experiment, *S. aureus* was diluted 1:6 (1.7 × 10^7^ CFU/mL) or 1:60 (1.7 × 10^6^ CFU/mL) in fresh TSB, respectively, for post-adhesion or a micro-pillar device. In *S. aureus* adhesion experiments, 1:60 (1.7 × 10^6^ CFU/mL) dilution was used.

### 2.3. IgG Depletion

The ACD-plasma of patients was collected in BD Vacutainer^®^ and maintained on ice during the procedures of depletion to avoid the activation of the complement. IgG depletion was obtained by passing human plasma-citrate at 10% diluted in TSB (3 mL) on a protein-G Sepharose^TM^ Fast Flow (GE Healthcare, Uppsala, Sweden) column, as indicated by the manufacturer’s instructions. Bound IgG were eluted with 0.1 M Glycine-HCl pH 2.8 and measured using the Pierce^TM^ Coomassie (Bradford reagent) protein assay kit (ThermoFisher Scientific, Waltham, MA, USA). The actual depletion of IgGs was evaluated by Western blot analysis after loading 1 µL/lane ACD-plasma on SDS-PAGE (10–12% acrylamide-bis; Bio-Rad Laboratories, Milan, Italy) and the use of horseradish peroxidase (HRP)-conjugated goat anti-human IgG (1 µg/mL; Jackson ImmunoResearch, West Grove, PA, USA).

### 2.4. IgG Titration

An indirect ELISA method with a 96-well plate coated with *S. aureus* lysate was used. *S. aureus* was cultured in TSB until O.D. = 0.6 A_600nm_, corresponding to 1 × 10^8^ CFU/mL. A total of 200 µL of the culture was resuspended in lysis buffer (150mM Tris-HCl pH 7.5 containing 2 mM EDTA, 2 mM EGTA, 1% triton X-100, all from Sigma/Merck, Germany, and a complete protease inhibitor cocktail from Roche, Basel, Switzerland). Lysate was obtained after three cycles of freezing and thawing. *S. aureus* lysate was diluted 1:100 in carbonate buffer (pH = 9.6, 35 mM NaHCO_3_, 15 mM Na_2_CO_3_) and incubated at 4 °C overnight for adsorption. The blocking of non-specific binding to plastic wells was performed with washing buffer containing 0.5% vol/vol Tween-20 in PBS^++^ pH 7.4, (0.9 mM CaCl_2_, 0.49 mM MgCl_2_, 137.9 mM NaCl, 2.7 mM KCl, 10 mM Na_2_HPO_4_, and 1.8 mM KH_2_PO_4_; Sigma-Aldrich, Darmstadt, Germany) with 5% dry milk (*w/v*) for 2 h at room temperature (r.t.). ACD-plasma was serially 10-fold diluted in washing buffer containing 100 µg/mL of purified goat IgGs (026202; #804535A, Invitrogen, Waltham, MA, USA) and then incubated on *S. aureus* lysate for 2 h at r.t. An anti-human IgG was used for the detection of specific anti-*S. aureus* IgGs (secondary antibody; goat anti-Human IgG HRP-conjugated, 1:5000 dil.; A18817 #9363092322, Invitrogen, Waltham, MA, USA). The addition of purified goat IgGs in the washing buffer avoids the IgGs binding activity of protein A of *S. aureus*. The results are expressed as [log] O.D at A_405nm_.

### 2.5. Microfluidic Device

Two different polydimethylsiloxane (PDMS) microfluidic devices were employed. PDMS is a commonly used material in microfluidics due to its flexibility, transparency, and ease of fabrication. Moreover, it can be a suitable substrate for studying biofilm formation and bacterial adhesion in various medical contexts [42]. The first microfluidic device was composed of 12 straight channels (16 mm long, 100 µm high and 800 µm wide) and was used to run experiments using different plasma and treatments after bacterial adhesion. The second device (micro-pillars chip) was composed of eight straight channels (40 mm long, 40 µm high and 1 mm wide) with five isolated micro-pillars (with a diameter of 50 µm) placed along the channel at 6 mm from each other and slightly shifted with respect to the midline of the channel. The presence of an isolated pillar acts to divert the flow from a rectilinear path, inducing secondary vortices that trigger the reproducible formation of filamentous biofilm structures known as streamers [43,44]. This device was used for experiments of *S. aureus* adhesion in flow and biofilm streamers formation. The devices were fabricated using soft lithography and rapid prototyping, as previously described [45]. Briefly, master molds were fabricated by patterning the negative photoresist SU-8 (Kayaku Advanced Materials, Inc., Westborough, MA, USA) on silicon wafers. Positive replicas of the microfluidic channels were obtained by pouring PDMS (Sylgard 184, Dow Corning, Midland, MI, USA) and a curing agent 10:1 (*w*/*w*) on the master and degassed in a vacuum chamber to remove bubbles. PDMS was thermally cured on a heat plate at 120 °C to create a negative replica mold. Cured PDMS was peeled off, and connecting holes (inlets and outlets) were created using a biopsy puncher (1.5 mm). The PDMS channels were irreversibly bonded to a glass slide upon treatment with oxygen plasma. The devices were sterilized by UV irradiation before each experiment.

### 2.6. Microfluidic Experimental Workflow and Live Cell Imaging

Microfluidic experiments were performed using TSB and different human plasma, serum and proteins, as described in the Section 3 and figure legend. ACD-plasma depleted from FG (FGˉ; FG-DP, #DP1-0097), Factor X (FXˉ; FX-DP, #DP10-0139), Factor VII (FVIIˉ; FVII-DP #DP7-0153), plasminogen (PLGˉ; PG-DP, #DP21-0025) and plasma control (NP; VisuConF UFNCP0125 #0019-73FCP) were acquired from Stago (Leiden, The Netherlands). Normal human serum (HS; NHS, #47c) was acquired from Complement Technology (Tyler, TX, USA). The recombinant (from NSO cells) purified human urokinase plasminogen activator (uPA; #HKY0921101) and human tissue-type plasminogen activator (tPA; #DATN032304) were acquired from R&D systems (Minneapolis, MN, USA). Fibrinogen (FG; human native fibrinogen plasminogen-depleted, 341578, #D00160002, Calbiochem, San Diego, CA, USA), fibronectin (FN; human native fibronectin; 341635, #3156097, Calbiochem, San Diego, CA, USA), hyaluronic acid (HA, hyaluronan from human umbilical cord; 385902, #B66144m Calbiochem, San Diego, CA, USA) and type I (#12CSP01A, Nutacon BV, Leimuiden, The Netherlands) and IV collagen (from equine tendon, 311501c, #L296/1, Biolife Italiana, Milan, Italy) were also used in specific experiments. Before each experiment, microfluidic channels were carefully filled with sterile TSB in order to prevent the entrance of air bubbles and to prime the observation chamber in the system. For each experimental condition, a glass syringe (1 mL, Inner Diameter 4.78 mm, BD Luer-Lok^™^) containing a different medium with or without bacteria was connected to the channel through needles 21 G (BD Microlance, 304432, BD, Milan, Italy) and Tygon tubings (inner diameter 508 µm, outer diameter 1.524 µm, #AAD04103, Saint-Gobain, France) and injected into the observation chamber. The flow was driven by a syringe pump (NE 1800, New Era Pump Systems, Farmingdale, NY, USA), using a flow rate of 0.5 μL/min. Syringes were kept in ice throughout the acquisition in order to avoid *S. aureus* growth in the syringe and to preserve the enzyme cascade efficacy (coagulation and fibrinolysis). As indicated, in specific experiments aimed at measuring *S. aureus* adhesion, microfluidic devices were precoated with the ECM components and incubated at 37 °C, 5% CO_2_ for 2 h. Microchannels were then injected with TSB containing *S. aureus* (1.7 × 10^6^ CFU/mL). In post-adhesion experiments using the straight 12-channel device, *S. aureus* (1.7 × 10^7^ CFU/mL) was initially seeded inside the microfluidic channels for 30 min at r.t. before starting the flow of different conditions of the medium and imaging acquisition. In experiments using a micro-pillar device, syringes containing *S. aureus* (1.7 × 10^6^ CFU/mL) and different conditions of the medium were used.

Propidium Iodide (PI) (#P4170, Sigma-Aldrich, Darmstadt, Germany) was added to a final concentration of 1 μg/mL to all the conditions and throughout the experiment as an elective probe for the detection of the eDNA associated with biofilm formation [44,46,47,48]. When indicated, the flow of plasma was trimmed up to 50 µL/min, and images were acquired every 5 min in order to evaluate the mechanical response of the biofilm colonies and the consequent *S. aureus* detachment from the surface of the device. All the experiments were performed under climate control (37 °C, 5% CO_2_ in a humidified atmosphere; Okolab, Naples, Italy). Images were acquired using two DMI8 Leica microscopy systems equipped with a 20 × air objective (20 ×/0.40NA HC PL FLUOTAR L). For each time frame, at least three to four consecutive but not-overlapping images for each channel and condition were acquired after sequential illumination with the Lumen 200 Fluorescence System (Prior Scientific Inc., Rockland, MA, USA) and the collection of the signal contribution for GFP (Em. 495/517 nm), PI (Em. 550/580 nm) and bright field contrast (BF) using an ORCA-Flash 4.0 V3 Digital CMOS camera (C13440-20CU; Hamamatsu, Milan, Italy). Images were acquired every 5 or 10 min up to 5 h using Leica Application Suite X software (LASX; v 3.5.5.19976) or Metamorph (v 7.10.1.161).

### 2.7. Analysis

Images were extracted from *.lif* files and renamed and processed using a custom Image-J/Fiji pipeline [49]. During the pre-processing steps, the images were downscaled to 1024 × 1024 pixels, and time-lapses were visually inspected in order to exclude critical acquisitions (e.g., bubble formation) and to ensure that images were in focus for the duration of the experiment. In general, measurements were carried out in regions of interest (ROIs) belonging to the central area of the channel in order to avoid borders where debris adhere and accumulate, and *S. aureus* grows unevenly as large clumps. For each experiment, a subset of at least three to four GFP images from different time points was used to train a pixel classification model using Ilastik (v 1.3.3 [50]) in order to distinguish GFP-positive (GFP^+^) regions from the background. The model was applied to segment all GFP images obtaining eight-bit masks. A custom pipeline using Image-J macro and R [51] was used to perform all the subsequent analysis. The mask was slightly enlarged (dilation of two pixels in each direction) to close small gaps before measuring the median fluorescence intensity (MFI) of the GFP raw signal inside the mask itself. For adhesion experiments, PI MFI was measured inside the mask at each time point, while in flow experiments, it was calculated above the manually thresholded background. In experiments using a micro-pillar device, a signal contiguous to the pillar was included in the analysis, and specific ROIs were created by hand using ImageJ. A minimal size of 200 µm^2^ was considered for the analysis. In the adhesion experiment, in order to reduce differences due to the initial seeding in the channels, as well as different background signal, both the GPF and PI signals were normalized to the initial MFI (relative MFI, rMFI).

In order to quantify the morphological differences between *S. aureus* colonies dependent on different growing conditions, we associated with each image its Circularity Index (CI) (4π area/perimeter^2^, range of 0–1). CI was calculated with the Analyze Particle plugin on the original non-dilated GFP mask, on colonies with sizes between 10 and 2000 µm^2^ and, typically, for up to 150 min in order to avoid the merging of adjacent colonies. Prism (GraphPad v. 9.5.1) was used to plot the data. The GFP^+^ area was calculated inside each ROI only on unmasked pixels. Statistical analysis was performed using non-parametric one-way ANOVA and a post hoc pairwise multiple comparisons test (Rstatix package kruskal_test and dunn_test functions). Non-parametric one-way ANOVA (Kruskal–Wallis test) on the different conditions was performed at each time point, and Dunn’s test was used to perform pairwise comparisons between selected groups. Initial time points (*t* < 100 min) were excluded from the statistical analysis on GFP MFI and PI MFI since, in most of the experiments, the increase in these quantities was negligible.

## 3. Results

### 3.1. Fibrinogen Is Essential in the Different Phases of S. aureus Biofilm Formation

The interaction of *S. aureus* with host molecules present in blood and ECM is reported to influence biofilm formation by promoting adhesion and aggregation [52]. We employed a multilayer method to define the relevance of ECM and hemostasis molecules in biofilm formation and evaluate the different stages that include attachment, colony formation and maturation, irreversible attachment or detachment (dispersal of *S. aureus* colonies), leading to the colonization of other sites [53,54,55].

**Figure 1:**
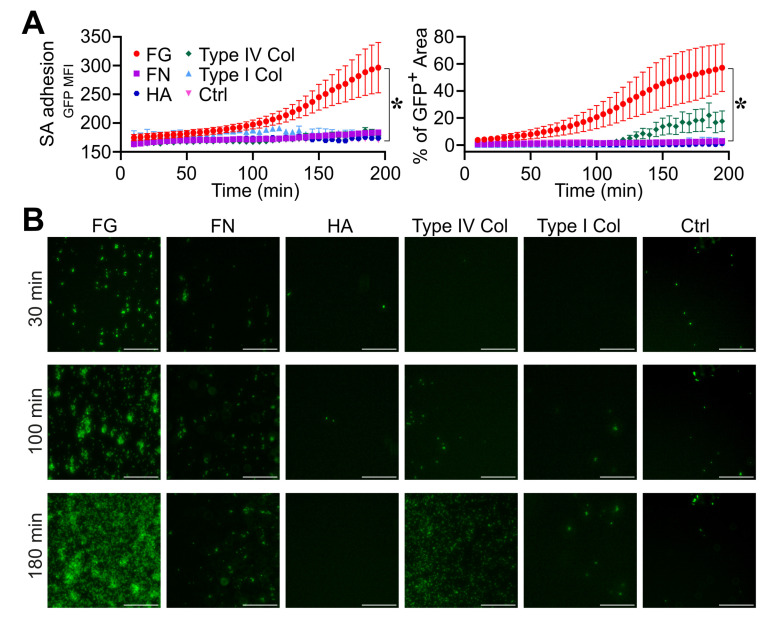
Role of fibrinogen in *S. aureus* (SA) adhesion. (**A**,**B**) The bottom surface of microfluidic channels coated with different ECM molecules was used. Fibrinogen (FG, 100 µg/mL), Fibronectin (FN, 100 µg/mL), Hyaluronic acid (HA, 100 µg/mL), type I collagen (100 µg/mL) and type IV collagen (100 µg/mL). Flux (0.5 µL/min) of *S. aureus* (1.7 × 10^6^ CFU/mL) in TSB. (**A**) GFP MFI ± SE and Mean % of GFP^+^ area ± SE overtime. Each point refers to the average of 3,6 ROIs (regions of interest) from n = 2 experiments performed. * *p <* 0.05 FG vs. ctrl (Dunn’s Test). (**B**) GFP images are shown at representative time points (*t* = 30, 100 and 180 min) referring to one experiment. Bar, 100 µm.

In the first series of experiments (n = 2), *S. aureus* adhesion was measured over time (0–200 min) on a glass bottom surface of straight channels of a microfluidic device previously adsorbed with purified FG, FN, HA and type I and type IV collagen (all at 100 µg/mL) (Figure 1 and Movies S1–S6). Among the molecules tested, FG was shown to be essential in inducing *S. aureus* adhesion to the surface, as ascertained by the quantification of GFP MFI (FG, 282 ± 91 vs. ctrl, 171 ± 1 at *t* = 180 min; n = 6, 2; *p* = 0.02) and the GFP^+^ area (FG, 53.5 ± 46% vs. ctrl, 0.4 ± 0.2% at *t* = 180 min; n = 6, 3; *p* = 0.02) (Figure 1A and Movie S1) compared with the control (Figure 1A and Movie S6). In the same experimental settings, no relevance was observed with the use of FN (Figure 1A and Movie S2), HA (Figure 1A and Movie S3), type I (Figure 1A and Movie S4) and type IV (Figure 1A and Movie S5) collagen-coated surfaces in *S. aureus* adhesion and growth; therefore, subsequent efforts in defining the role of host molecules in the stages leading to *S. aureus* biofilm formation focused on FG.

In a different series of experiments (n = 4), *S. aureus* was seeded on the surface of a microfluidic device, and the effect of FG in the assembly of the biofilm matrix was assessed by flowing (with a flow rate of 0.5 µL/min) normal human plasma (NP) in comparison with FG-depleted ACD-plasma (FGˉ). As established in a previous setup (Figure S1A,B and Movies S7–S11), the use of NP ensured the effective visualization and measurement of biofilm compared to human serum (HS) used at equal % (PI rMFI: 10% NP, 2.4 ± 0.4 vs. HS, 1.0 ± 0.1 at *t* = 180 min; n = 5, 3; *p* = 0.03) (Figure S1A,B and Movies S7 and S9). This suggests the relevance of FG and of an active coagulation cascade in biofilm formation, since these elements are consumed during serum preparation [56] (n = 3 NP, levels of FG = 230.7 ± 53.5 mg/dl vs. undetectable in correspondent HS; not shown). Similar *S. aureus* growth associated with biofilm detection was observed when percentages of 1, 3 and 10 NP were used (GFP rMFI: 10%, 7.8 ± 1.8, 3%, 6.0 ± 0.2 1%, 7.0 ± 1.1, at *t* = 180 min; PI rMFI: 10%, 2.1 ± 0.1, 3%, 2.29 ± 0.2, 1%, 1.5 ± 0.1, at *t* = 180 min; n = 8, 2, 3) (Figure S1A,B and Movies S9–11). However, *S. aureus* colonies showed typical characteristics when grown in 10% plasma compared to the other conditions, and, as ascertained from morphometric measurement parameters (Figure S1C), they appeared more expanded and rounded (CI: 10%, 0.78 ± 0.04, 3%, 0.76 ± 0.01, 1%, 0.68 ± 0.02, at *t* = 100 min; *p* = 0.03 NP 10% vs. NP 1%; n = 8, 3), indicating a mature state of *S. aureus* colonies associated with the highest biofilm measurement.

**Figure 2:**
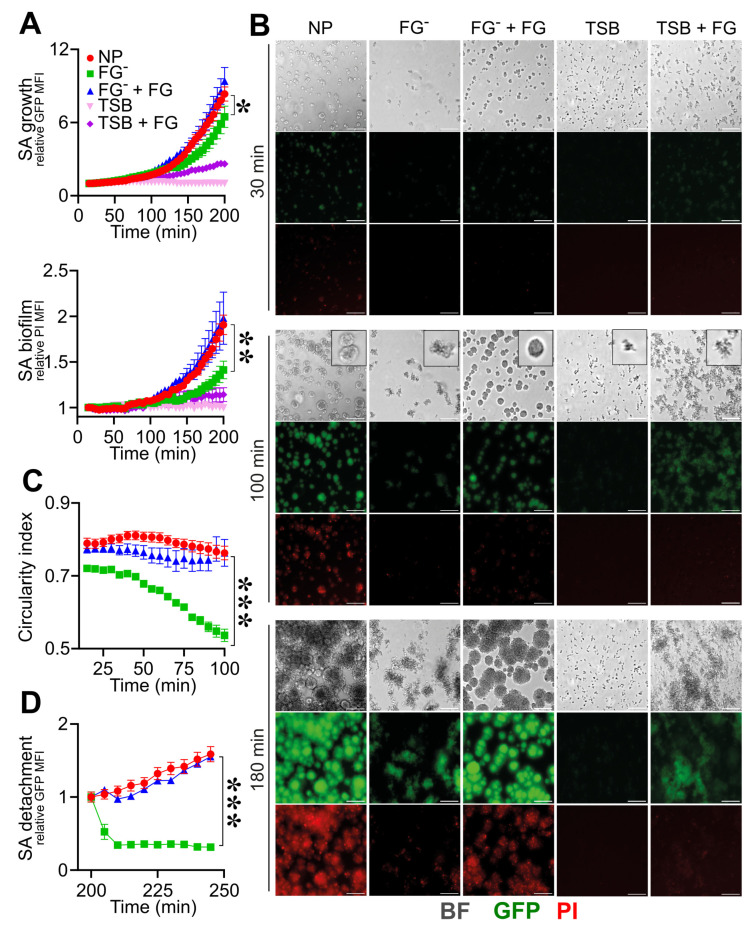
Role of fibrinogen in the assembly and formation of *S. aureus* (SA) biofilm. (**A**–**D**) *S. aureus* (1.7 × 10^7^ CFU/mL) previously adhered on the bottom surface of straight microfluidic channels. Conditions include TSB, 10% of normal (NP) and fibrinogen-depleted (FGˉ) ACD-plasma diluted in TSB and FGˉACD-plasma diluted in TSB added with human purified FG (400 µg/mL) and FG (400 µg/mL) in TSB. (**A**) *S. aureus* growth and biofilm formation were evaluated, respectively, as relative GFP (upper) and PI (lower) MFI. Values are represented as functions of time and normalized over the first time point. The PI signal was considered superimposed to the GFP-positive mask, as described in Materials and Methods. * *p <* 0.05, ** *p <* 10^−3^ FGˉ vs. NP at *t* = 180 min, n = 12, 15, Dunn’s test. (**B**) Images of bright field (BF), GFP and PI at representative time points (*t* = 30, 100 and 180 min), referring to one experiment. At *t* = 100 min, BF close-up images representing *S. aureus* morphology are also shown. Bar, 50 µm. (**C**) Mean CI of *S. aureus* colonies *** *p <* 10^−4^ FGˉ vs. NP at *t* = 100 min. (**D**) Relative GFP MFI over time after the flux increase (at *t* = 200 min, 10 µL/min for 30 min to 50 µL/min until the end of the experiment). *** *p <* 10^−4^ FGˉ vs. NP, n = 9, 12, Dunn’s test. (**A**,**C**) Mean ± SE of 9 to 15 ROIs from three experiments out of four performed with similar results. (**D**) Mean ± SE of 3 to 12 ROIs from two experiments out of four performed with similar results.

As shown in Figure 2A,B, the use of FGˉ was associated with decreased *S. aureus* growth and biofilm formation (GFP rMFI: 4.6 ± 2.2; PI rMFI: 1.2 ± 0.2, at *t* = 180 min; n = 12; *p* = 0.02) when compared to NP (GFP rMFI: 6.2 ± 1.8; PI rMFI: 1.6 ± 0.2, at *t* = 180 min; n = 15) (Figure 2A,B and Movies S12 and S13). Moreover, *S. aureus* colonies in FGˉ appeared disorganized and unrounded compared to NP (CI: 0.76 ± 0.07, NP vs. 0.54 ± 0.06, FGˉ; at *t* = 100 min; n = 15, 12; *p* = 1 × 10^−5^) (Figure 2B,C and Movies S12 and S13), indicating a role for FG in the assembly of a stable matrix essential for colony growth, stability and biofilm formation. On the line, a progressive increase in the flow rate up to 50 µL/min resulted in a detachment of *S. aureus* colonies in FGˉ (GFP rMFI: 0.3 ± 0.1, at *t* = 240 min relative to *t* = 200 min; n = 9; *p* = 10^−4^) compared to NP (GFP rMFI: 1.5 ± 0.1, at *t* = 240 min relative to *t* = 200 min; n = 12), which appeared to resist stably (Figure 2D and Movies S12 and S13). The reconstitution of FGˉ at physiological levels with human purified FG (400 µg/mL) rescued defects of *S. aureus* growth (GFP rMFI: 6.8 ± 2.1, at *t* = 180 min; n = 6; *p* = 0.02 vs. FGˉ) (Figure 2A,B and Movie S14), the aspect and organization (CI: 0.76 ± 0.11 at *t* = 100 min; n = 9; *p* = 4 × 10^−4^ vs. FGˉ) and the stability of colonies (GFP rMFI, 1.5 ± 0.1, at *t* = 240 min relative to *t* = 200 min; n = 6; *p* = 0.01 vs. FGˉ) (Figure 2C,D and Movie S14) and biofilm formation (PI rMFI, 1.7 ± 0.4, at *t* = 180 min; n = 6; *p* < 0.05 vs. FGˉ) (Figure 2A,B and Movie S14) observed in conditions of FG-deficit. The addition of purified FG in TSB was associated with increased *S. aureus* adhesion and growth over time (GFP rMFI: FG + TSB, 2.36 ± 0.8 vs. TSB 1.0 ± 0.1, at *t* = 180 min; n = 16, 9; *p* < 10^−4^). However, FG alone was not sufficient to recapitulate biofilm formation compared to plasma (PI MFI, FG + TSB, 1.14 ± 0.3 vs. NP, at *t* = 180 min; n = 16; *p* < 10^−5^) (Figure 2A,B and Movies S15 and S16), thus indicating that the *S. aureus* coagulases-mediated initiation of the coagulation cascade and fibrin formation is essential in supporting biofilm establishment. The use of plasma depleted molecules of hemostasis, emphasizing a close dependence on *S. aureus*-mediated fibrin in biofilm formation, thus excluding a role in the host coagulative response. Indeed, as shown in Figure S2, similar *S. aureus* growth and biofilm formation were observed in FVII- (FVIIˉ) and in FX- (FXˉ)-depleted plasma and NP, in agreement with *S. aureus* coagulases’ capacity to convert FG to fibrin through prothrombin activation, without involving host coagulation [55].

**Figure 3:**
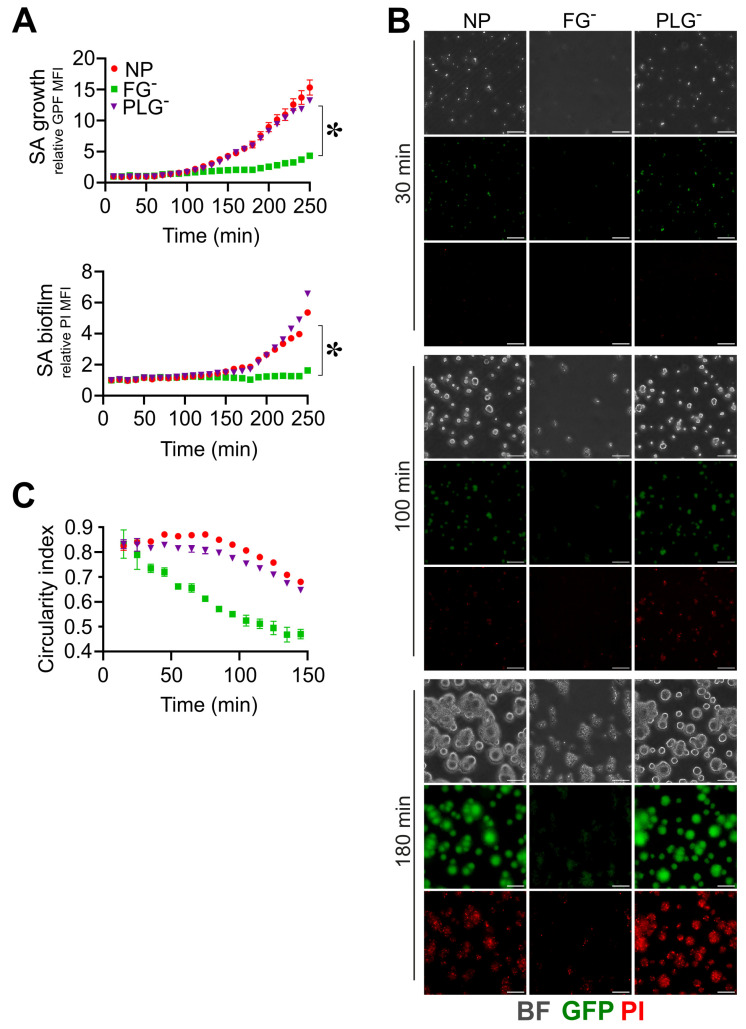
Plasminogen does not affect *S. aureus* (SA) biofilm formation. (**A**–**C**) Same experimental setting and analysis as those in Figure 2 were used; 10% of PLG-depleted (PLGˉ) or FGˉ and NP ACD-plasma diluted in TSB were used. (**A**) Relative GFP (upper) and PI (lower) MFI ± SE over time. * *p <* 0.05 PLGˉ vs. FGˉ, *t* = 180 min, Dunn’s test. (**B**) BF, GFP and PI images at *t* = 30, 100 and 180 min, representative of one experiment. Bar, 50 µm. (**C**) Mean CI of *S. aureus* colonies. (**A**,**C**) Each point refers to the Mean ± SE of three ROIs from one experiment of three performed.

The staphylokinase-dependent activation of PLG prevents biofilm formation in a murine model of catheter infection [25]. Therefore, a possible relevance of active PLG in interfering with biofilm formation was evaluated using PLG-depleted plasma (PLGˉ). *S. aureus* showed similar growth and biofilm formation in PLGˉ and NP (GFP rMFI, 6.1 ± 1.0 vs. 6.0 ± 0.2; PI rMFI, 1.9 ± 0.1 vs. 1.7 ± 0.1, at *t* = 180 min; n = 3) (Figure 3A,B and Movies S17 and S19), whereas, as expected, *S. aureus* growth and biofilm were abolished in FGˉ (GFP rMFI, 2.1 ± 0.1; PI rMFI, 1 ± 0.1 vs. at *t* = 180 min; n = 3; *p* = 0.02 vs. PLGˉ) (Movie S18). A similar morphology and structure of *S. aureus* colonies were also observed in PLGˉ and NP (CI, 0.75 ± 0.02, vs. 0.81 ± 0.01, at *t* = 180 min; n = 3) (Figure 3B,C and Movies S17–S19); thus, in our experimental approach, no relevance of PLG in altering the biofilm process was observed.

**Figure 4:**
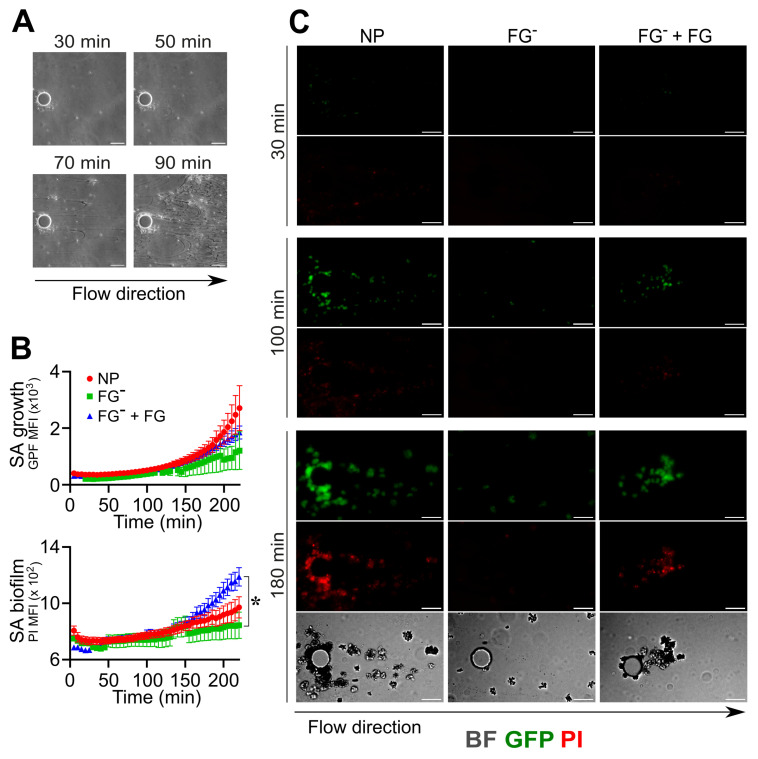
Role of fibrinogen in the adhesion, assembly and formation of biofilm by *S. aureus* (SA) in flow. (**A**–**C**) A pillar-based microfluidic device was used. *S. aureus* (1.7 × 10^6^ CFU/mL) was injected with a flow rate of 0.5 µL/min at the same experimental conditions as in Figure 2. (**A**) Representative BF images which refer to one experiment of six performed, showing different initiation phases (adhesion, coagulation, fibrin matrix assembly) that lead to biofilm formation around the micropillar. (**B**) *S. aureus* growth as GFP MFI (×10^3^) and biofilm detection as PI MFI (×10^3^) as a function of time. Each point refers to the Mean ± SE of 6–15 ROIs from three experiments. Missing values in FGˉ correspond to time points where less than two pillars (over six) had a detectable signal, as described in the Section 2. * *p <* 0.05 FGˉ + FG vs. FGˉ at *t* = 180 min, Dunn’s test. (**C**) GFP, PI and contrast images are shown at *t* = 30, 100 and 180 min. Bar, 50 µm. (**A**,**C**) Arrow indicates the flow direction.

In order to corroborate the results obtained by further mimicking a pathological contest of infection occurring in an indwelling medical device [57], experiments (n = 3) using the same conditions were performed by flowing *S. aureus* into a microfluidic device equipped with micro-pillars (Figure 4). In NP, *S. aureus* bacteria in flow adhere to the inner surfaces of the microfluidic channels, aggregate and form a coagulative state close to the pillar, which is essential in supporting the formation of filamentous biofilm structures known as “streamers” (Figure 4A–C and Movie S20) [44,58]. In FGˉ, *S. aureus* was unable to perform the first phases involving adhesion, aggregation and coagulation (GFP MFI: FGˉ, 882 ± 668 vs. NP, 1311 ± 724 at *t* = 180 min; n = 6,10; 2 field with a detectable signal in FGˉ); therefore, biofilm formation was not detected under these conditions (Figure 4B,C and Movie S21). Interestingly, the reconstitution of FG (400 µg/mL) alone in FGˉ rescued the *S. aureus*’s ability to adhere to the pillar, aggregate (GFP MFI: FGˉ + FG, 1212 ± 571, at *t* = 180 min; n = 15; 15 field with a detectable signal), coagulate and form biofilm (PI MFI: FGˉ + FG, 986 ± 187, FGˉ 809 ± 148 at *t* = 180 min; n = 15, 4; *p* = 0.03) (Figure 4B,C and Movie S22).

Therefore, as assessed by employing two different microfluidic methods, FG plays an essential role in triggering early phases of *S. aureus* biofilm formation and by driving adhesion and aggregation to the surface. *S. aureus*-induced fibrin polymerization is critical in the assembly and organization of the matrix, leading to biofilm constitution.

### 3.2. Triggering Fibrinolysis Interferes in the Formation of S. aureus Biofilm

**Figure 5:**
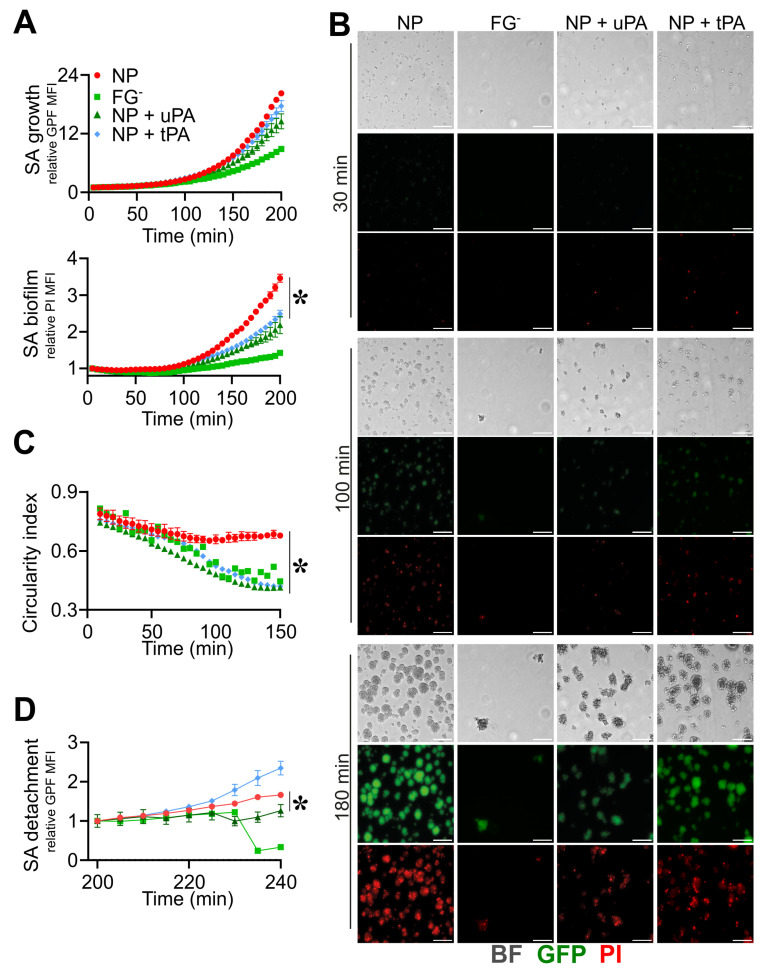
Triggering fibrinolysis interferes in the formation of *S. aureus* (SA) biofilm. (**A**–**D**) Straight microfluidic channels, with *S. aureus* (1.7 × 10^7^ CFU/mL) previously adhered on the bottom surface, were used in the experiments (n = 2); 10% of NP with or without the addition of recombinant purified uPA (0.4 µg/mL) or tPA (0.4 µg/mL) and FGˉ were used. (**A**) *S. aureus* growth as relative GFP MFI ± SE (upper) and biofilm formation as PI MFI ± SE (lower) over the initial time. (**B**) BF, GFP and images referring to one representative experiment are shown at *t* = 30, 100 and 180 min. Bar, 50 µm. (**C**) CI of *S. aureus* colonies expressed as the Mean ± SE. (**D**) *S. aureus* detachment as an expression of the relative GFP after the flow rate was increased up to 10 µL/min for 30 min and later to 50 µL/min until the end of the acquisition. Each point is the Mean ± SEM of three to eight ROIs from one experiment out of two performed with similar results. * *p <* 0.05 NP vs. NP + uPA at *t* = 180 min (**A**), *t* > 100 min (**C**), *t* = 240 min (**D**), Dunn’s test.

The use of fibrinolytic agents, such as streptokinase [59], nattokinase and trypsin-like enzymes [60], in combination with antimicrobials, was efficient in *S. aureus* device-related infection in vivo. A prophylaxis use of the tissue-type plasminogen activator (tPA) as a coating of the implant surface prevented *S. aureus* adhesion and increased the susceptibility to treatment [61], possibly as a consequence of the increased recruitment and activation of PLG, hence favoring fibrin digestion. This evidence prompted us to define the role of the fluid-phase activation of fibrinolysis in the phases of the biofilm process using both tPA and urokinase plasminogen activator (uPA). As shown in Figure 5, the addition of uPA (0.4 µg/mL) and tPA (0.4 µg/mL) in NP reduced biofilm formation throughout the experiments (PI rMFI: uPA, 1.8 ± 0.3, tPA, 2.0 ± 0.1, NP, 2.7 ± 0.1, at *t* = 180 min; n = 9, 8, 3; *p* = 0.01, *p* = 0.03 uPA, tPA vs. NP) without affecting *S. aureus* growth (GFP rMFI: uPA, 10.5 ± 3.0, tPA, 12.5 ± 1.6, vs. NP, 14.0 ± 0.3, at *t* = 180 min; n = 9, 8, 3; *p* = 0.04 uPA vs. NP) (Figure 5A,B and Movies S23–S26). The appearance of *S. aureus* colonies in the presence of both uPA and tPA was reminiscent of the disorganized, non-rounded morphology observed in conditions of FG-deficiency (CI: uPA, 0.48 ± 0.02, tPA, 0.52 ± 0.02, FGˉ, 0.53 ± 0.02, NP, 0.66 ± 0.04, at *t* = 100 min; n = 8, 3; *p* = 0.02 uPA, tPA vs. NP) (Figure 5B,C and Movie S24). Moreover, when subjected to a progressive increase in the flow rate (up to 50 µL/min), colonies treated with uPA showed increased detachment compared with NP (GFP rMFI: uPA, 1.26 ± 0.16, vs. NP, 1.66 ± 0.05, at *t* = 240 min; n = 8, 3; *p* = 0.03) (Figure 5D), whereas an increase in colony detachment was appreciated using tPA (Movie S26).

**Figure 6:**
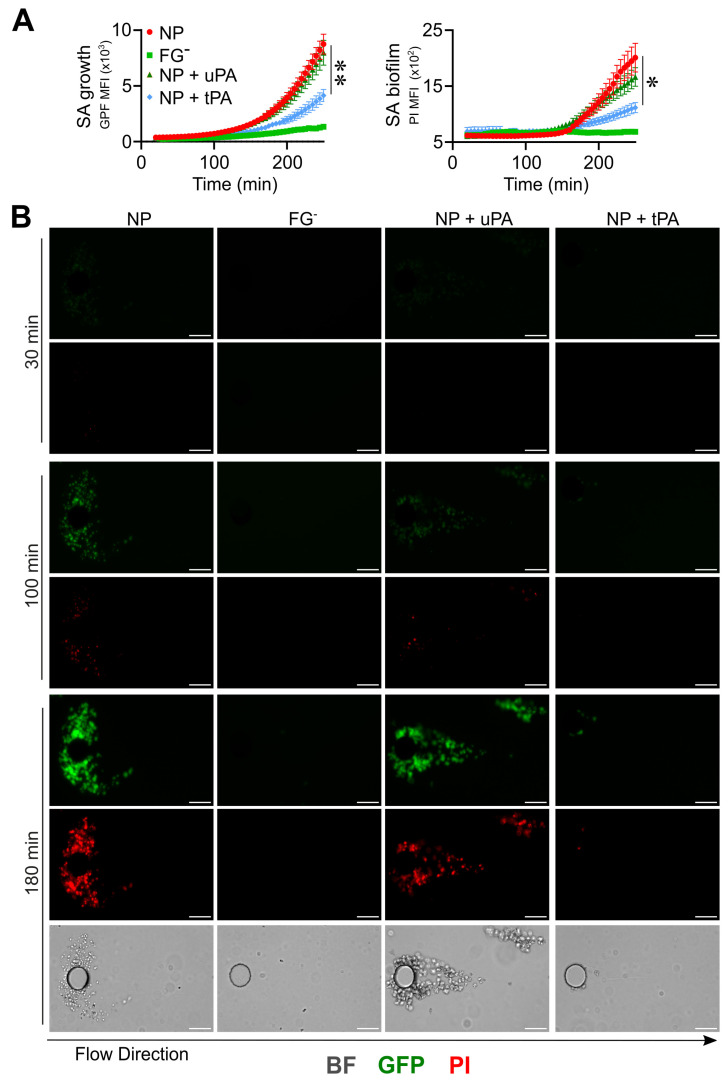
Triggering fibrinolysis interferes in the initial phase, leading to biofilm formation by *S. aureus* (SA) in flow. (**A**,**B**) A pillar-based microfluidic device and the same experimental conditions as those in Figure 4 were used; 10% of NP with or without the addition of recombinant purified uPA (0.4 µg/mL) or tPA (0.4 µg/mL) and FGˉ were used. (**A**) *S. aureus* growth (left) as GFP MFI (×10^3^) and biofilm detection (right) as PI MFI (×10^3^) as a function of time. Each point refers to the Mean ± SE of 8–10 ROIs from two experiments. * *p <* 0.05, ** *p <* 0.005 NP vs. NP + tPA, n = 10, *t* = 220 min. (**B**) GFP, PI and contrast images (*t* = 30, 100 and 180 min) of one representative experiment. Bar 50 µm. The arrow indicates the flow direction.

In pillar-based devices, similar results were obtained, with tPA showing a higher efficiency than uPA, in line with its high specificity of activating fibrinolysis in the fluid phase compared to the cell-mediated fibrinolysis at tissue sites [62]. Indeed, in the presence of tPA, uPA and NP, the PI MFI values were 965 ± 230, 1391 ± 466 and 1542 ± 628 (*t* = 220 min; n = 10; *p* = 0.04), (Figure 6A,B and Movies S27, S29 and S30). The use of tPA resulted in *S. aureus*’s inability to trigger the initial actions critical in biofilm formation, such as adhesion, aggregation (GFP MFI, tPA, 2639 ± 1328 vs. NP, 5644 ± 1870, at *t* = 220 min; n = 10; *p* = 0.002) and clot formation essential in the continuation of the process (Figure 6A,B and Movies S27, S29 and S30). In both methods, FGˉ was used as a reference control of the growth, biofilm production and morphology of *S. aureus* colonies (Figure 5A–D and Figure 6A,B and Movies S24 and S28).

The results indicate a role of fibrinolysis in inhibiting *S. aureus* biofilm formation, possibly by acting on the degradation of fibrin. In agreement with the results obtained in FG-deficiency, the activation of fibrinolysis alters *S. aureus* adhesion and growth, thus interfering from the earliest stages in the biofilm constitution.

### 3.3. The Reactivation of Fibrinolysis in S. aureus-Induced Sepsis Favors an IgG-Mediated Immune Response

In *S. aureus* biofilm, a fibrin scaffold provides high resistance to antimicrobial treatments and immune cell recognition [63]. In blood, *S. aureus* coagulases are essential to forming a mechanical barrier to protect *S. aureus* from recognition by opsonins [23]. In *S. aureus*-induced septicemic patients, an impairment of fibrinolysis contributes to disseminated intravascular coagulation (DIC) [11,64,65,66]. Therefore, we evaluated the effect of fibrinolysis reactivation in the immune response in *S. aureus*-induced septic patients.

**Figure 7:**
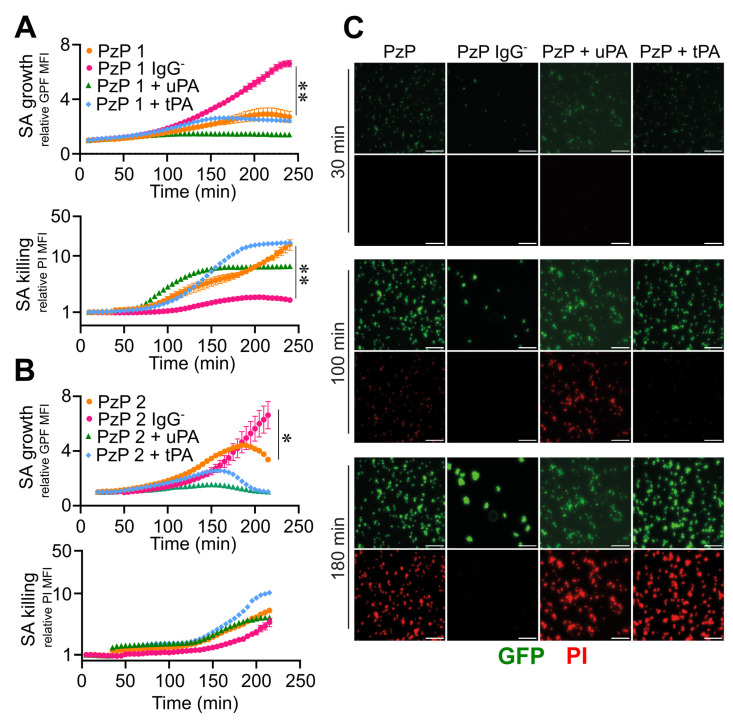
Reactivation of fibrinolysis in *S. aureus*-induced sepsis favors IgG-mediated pathogen killing. (**A**–**C**) Microfluidic channels and *S. aureus* (SA) (1.7 × 10^7^ CFU/mL) that were previously adhered were used; 10% of ACD-plasma of PzP 1 (higher titer of IgG anti-SA) (**A**) and PzP 2 (lower titer of IgG anti-SA) (**B**) in combination with uPA (0.4 µg/mL) or tPA (0.4 µg/mL) diluted in TSB were fluxed (0.5 µL/min). PzP 1 and PzP 2 ACD-plasma depleted of IgGs (IgGˉ) was also used to ascertain an anti-*S. aureus* IgG-mediated pathogen killing. (**A**,**B**) Upper: SA growth plotted as relative GFP MFI ± SE as a function of time. (**A**,**B**) Lower: *S. aureus* killing as PI MFI ± SE, considered superimposed to a GFP-positive mask, as described in the Section 2. A log scale was used to improve data visualization. (**A**,**B**) Each point refers to three to eight ROIs from one experiment (PzP 1) and three to eight ROIs from one experiment (PzP 2). (**A**,**B**) * *p <* 0.05 PzP 2 vs. PzP 2 IgGˉ (n = 3, 4); ** *p <* 0.01 PzP 1 vs. PzP 1 IgGˉ (n = 4, 8), at *t* = 220 min, Dunn’s test. (**C**) GFP and PI images of an experiment involving PzP 1 at *t* = 30, 100 and 180 min. Bar, 50 µm.

In this regard, *S. aureus* that was previously adhered on the bottom of straight channels was flushed with 10% plasma obtained from patients with a bacteriological diagnosis of blood *S. aureus* infection and a different titre of specific anti-*S. aureus* IgGs, high in ACD-plasma from patient 1 (PzP 1) and low in ACD-plasma from patient 2 (PzP 2) (Figure S3). As shown in Figure S1, the method and analysis were revised in detail in order to specifically distinguish the detection of biofilm formation from pathogen killing, as expected from an incubation of *S. aureus* in a context of specific immunoresponsiveness. Actually, it was possible to obtain an unbiased measure of *S. aureus* biofilm formation in NP vs. *S. aureus* killing (vancomycin-mediated in Figure S1D–F). Therefore, at increased PI MFI, GFP MFI correspondingly decreased due to bacterial death (Figure S1E), as ascertained by the relative GFP MFI values (NP, 7.8 ± 1.8, vs. vancomycin, 1.8 ± 0.3, at *t* = 180 min; n = 8, 4; *p* = 0.007) of *S. aureus* growth and relative PI MFI (NP, 2.2 ± 0.5, vs. vancomycin, 4.8 ± 1.1, at *t* = 180 min; n = 8, 4; *p* = 0.007) (Figure S1D,E and Movies S31 and S32). IgG depletion from both plasmas (PzP 1 and PzP 2) resulted in a decrease in the relative PI MFI (PzP 1 IgG^ˉ^, 1.7 ± 0.1; PzP 2 IgG^ˉ^, 1.8 ± 0.2 at *t* = 180 min; n = 8, 4; *p* = 0.007, *p* = 0.03) and an increase in the relative GFP MFI (PzP 1 IgG^ˉ^, 4.3 ± 0.4, at *t* = 180 min; n = 4; *p* = 0.007; PzP 2 IgG^ˉ^, 4.2 ± 1.4, at *t* = 180 min; n = 4) compared to the control (PI rMFI: 4.6 ± 1.4, PzP 1, 3.0 ± 0.4 PzP 2; GFP rMFI: 2.6 ± 0.9, PzP 1, 4.3 ± 0.2, PzP 2, at *t* = 180 min; n = 8, 3) (Figure 7A–C and Movies S33 and S34), indicating the IgG-mediated killing of *S. aureus*. The activation of fibrinolysis by the addition of uPA and tPA promoted earlier *S. aureus* killing (range of time: PzP 1 from *t* = 75 min, PzP 2 from *t* = 120 min), correlated with the presence of higher (PzP 1) or lower (PzP 2) levels of anti-*S. aureus* IgGs (Figure 7A–C and Movies S35 and S36).

Therefore, in *S. aureus*-induced sepsis, the reactivation of fibrinolytic activity promotes the clearance of an *S. aureus*-associated surface, thereby impairing biofilm formation.

## 4. Discussion

The participation of ECM elements in the innate immune response is an ongoing topic [16]. Although generally considered to be separate from the innate immune response, the evasion of pathogens from the host defense involves mechanisms that are mediated by their interaction with the ECM as well as by the manipulation of elements of hemostasis [66]. In specific contexts, the interaction of pathogens with the same molecules is a disadvantage for the onset of infection. In fact, ECM elements act as an integral part of inflammatory and innate immune responses [67,68], have antimicrobial functions by acting as recognition elements and behave as osponins [15,69]. On the other hand, fluid phase molecules of innate immunity play essential roles in tissue repair and healing through remodelling the ECM by interacting with its elements [12] or regulating the activities of the cells involved [70]. A mutually functional relationship between hemostatic and innate immune responses is consolidated [1,2].

In the present study, we investigated the complexity of interactions among the ECM and hemostatic system in the biofilm formation of *S. aureus*, a leading cause of hospital-associated bloodstream infections and the most common cause of several life-threating conditions, such as endocarditis and sepsis [18,19,21]. *S. aureus* accounts for the majority of medical device-associated infections and sepsis, which are difficult to treat because of the biofilm structure that allows them to evade the immune response and disfavor the efficacy of antibiotic treatment [28].

By investigating the underlying mechanisms of these processes, we sought to develop insights that could contribute to improving medical treatments. To this end, we have used Live Cell Imaging, microfluidics and data analysis to mimic an in vivo pathological context of *S. aureus* infection on an indwelling medical device. Specifically, we exploited different microfluidic channels to capture the spatiotemporal dynamics of *S. aureus* formation and to evaluate the effect of ECM and hemostasis molecules in this process. This parallel experimentation enabled efficient data collection and reduced experimental bias, as it allows for the comparison of experimental conditions within the same analysis. In addition, we used a newly developed microfluidic device whose basic unit is a straight channel with isolated micropillars located along its length [37,43]. The pillars serve as nucleation sites for the formation of streamers [43,44]. The induction of streamer formation provides a valuable approach to probing the composition and mechanical properties of a biofilm [43].

*S. aureus*-driven molecular mechanisms at the various phases leading to the formation of the biofilm have been extensively investigated [71]. On the other hand, there is scattered evidence on the role of host’s molecules in influencing the biofilm process. Surface-associated host proteins on implants are reported to mediate the adhesion of *S. aureus* [52], and evidence points to a functional relationship between *S. aureus*, hemostasis elements and ECM in the initiation of biofilm [13,36]. The broad-spectrum approach of the different ECM molecules used in our study allowed for consolidating the essential role of fibrinogen in promoting *S. aureus* adhesion to the device surface, thus allowing for the initiation of the process of biofilm formation, with respect to the other molecules considered in the study. Moreover, in line with reported evidence [24,25], fibrin conversion is essential in assembling a stable matrix supporting biofilm formation and providing the attachment and resistance of colonies to fluid flow. At this stage, we demonstrated a functional exclusivity of *S. aureus*-driven coagulation, since no effect was observed in experiments using human plasma depleted of coagulation elements.

The formation of a stable and impenetrable protective coagulative matrix underlies the altered recognition by immune system molecules and the ineffective treatment of biofilm-associated *S. aureus* [33,72,73]. Evidence suggest an effect of fibrinolytic agents (e.g., streptokinase, nattokinase) in preventing *S. aureus* biofilm formation in vitro and enhancing the efficacy of antimicrobials in *S. aureus* device-related infections in vivo [59,60]. A prophylaxis use of tPA, as a coating of devices, prevented *S. aureus* adhesion and increased susceptibility to the treatment in vitro [61]. Using two different microfluidics systems, we defined the role of uPA- and tPA-mediated fibrinolysis activation in plasma in the inhibition of biofilm formation at the different stages that include attachment, colony formation and maturation, thus expanding the understanding of the mechanisms underlying the activity of these mediators. In the multichannel platform, an inhibitory effect of uPA-mediated fibrinolysis activation has never been described. Using the pillar-based device, the activation of tPA-mediated fibrinolysis resulted in interference in *S. aureus* adhesion, aggregation and coagulation and hence in the continuation of the process of biofilm formation.

Hereafter, the same rationale prompted us to evaluate the effect of fibrinolysis in promoting *S. aureus* recognition and immune effector functions favored by the remodeling of the fibrin matrix interacting with *S. aureus* adhering to device surfaces. In *S. aureus*-induced septicemic patients, the hemostatic system is activated in a dysregulated manner due to the alteration of a pro/anticoagulant system and the impairment of fibrinolysis (the so-called fibrinolytic shut-down), contributing to multiorgan damage and mortality [11,64,65]. Indeed, plasminogen activator inhibitor-1 (PAI-1) is a crucial regulator of fibrinolysis [74]. Increased levels of PAI-1 in sepsis predict disease severity and mortality [75], thus indicating a role of fibrinolysis in the disease outcome by inhibiting the disseminated thrombotic process in the microcirculation, a major cause of multiple-organ dysfunction in sepsis [76,77]. Thus, our results obtained through approaches that mimic the in vivo condition indicate that, despite the presence of antibodies in septicemic individuals [78], a reduction in fibrinolysis that promotes biofilm formation can alter an immune-mediated recognition and clearance of *S. aureus*. We observed that the activation of fibrinolysis is associated with the prevention of *S. aureus* biofilm formation and the enhancement of IgG-mediated pathogen killing, most likely associated with fibrinolysis activity in degrading *S. aureus*-associated fibrin. This translational part deserves extensive insights and will be extended to an increased number of patients and to associative analyses related to the clinical outcome by evaluating markers of hemostasis, inflammation and disease severity. Future studies will also be aimed at evaluating a synergistic effect between fibrinolysis activation and innate defense systems such as the complement system and other fluid-phase mediators of innate immunity in response to biofilm-associated bacteria.

## 5. Conclusions

*S. aureus*-forming biofilm is a cause of critical infections, and this represents a medical challenge given the lack of therapeutic approaches. Our study defines the importance of host-derived hemostasis factors in the different stages leading to *S. aureus*-driven biofilm formation, highlighting a central role of fibrinolysis in preventing biofilm initiation and formation. Actually, fibrinolysis is effective in unmasking surface-associated *S. aureus* and, in turn, in reactivating recognition and effector functions of the immune system, leading to pathogen clearance. The results are therefore instrumental in the development of new combined therapeutic approaches for the clinical management of *S. aureus* biofilm-associated infections and sepsis.

## Data Availability

All data needed to evaluate the conclusions in the paper are present in the paper and/or in the Supplementary Materials. Additional data related to this paper may be requested from the corresponding authors.

## Supplementary Materials

The following supporting information can be downloaded at: https://www.mdpi.com/article/10.3390/pathogens12091141/s1, Figure S1: Development of the imaging and microfluidics combined method for assessing *S. aureus* adhesion, growth and biofilm formation. Figure S2: Irrelevance of host coagulation elements in *S. aureus* biofilm formation. Figure S3: Detection of anti-*S. aureus* IgGs in plasma from septicemic patients. Table S1: List of abbreviations. Movies S1–S6: Role of fibrinogen in *S. aureus* adhesion (MOVIE_S1_Fig1_FG.mp4; MOVIE_S2_Fig1_FN.mp4; MOVIE_S3_Fig1_HA.mp4; MOVIE_S4_Fig1_TypeIV_Col.mp4; MOVIE_S5_Fig1_TypeI_Col.mp4; MOVIE_S6_Fig1_Ctrl.mp4). Movies S7–S11: Development of the imaging and microfluidics combined method for assessing S. aureus adhesion, growth and biofilm formation (MOVIE_S7_FigS1_HS10%.mp4; MOVIE_S8_FigS1_HS30%.mp4; MOVIE_S9_FigS1_NP10%.mp4; MOVIE_S10_FigS1_NP3%.mp4; MOVIE_S11_FigS1_NP1%.mp4). Movies S12–S16: Role of fibrinogen in the assembly and formation of S. aureus biofilm (MOVIE_S12_Fig2_NP.mp4; MOVIE_S13_Fig2_FG-.mp4; MOVIE_S14_Fig2_FG- +FG.mp4; MOVIE_S15_Fig2_TSB.mp4; MOVIE_S16_Fig2_TSB+FG.mp4). Movies S17–S19: Plasminogen does not affect S. aureus biofilm formation. (MOVIE_S17_Fig3_NP.mp4; MOVIE_S18_Fig3_FG-.mp4; MOVIE_S19_Fig3_PLG-.mp4). Movies S20–S22: Role of fibrinogen in the adhesion, assembly and formation of biofilm by S. aureus in flow (MOVIE_S20_Fig4_NP.mp4; MOVIE_S21_Fig4_FG-.mp4; MOVIE_S22_Fig4_FG- +FG.mp4). Movies S23–S26: Triggering fibrinolysis interferes in the formation of S. aureus biofilm (MOVIE_S23_Fig5_NP.mp4; MOVIE_S24_Fig5_FG-.mp4; MOVIE_S25_Fig5_upa.mp4; MOVIE_S26_Fig5_tPA.mp4). Movies S27–S30: Triggering fibrinolysis interferes in the initial phase, leading to biofilm formation by S. aureus in flow (MOVIE_S27_Fig6_NP.mp4; MOVIE_S28_Fig6_FG-.mp4; MOVIE_S29_Fig6_upa.mp4; MOVIE_S30_Fig6_tPA.mp4). Movies S31–S32: Development of the imaging and microfluidics combined method for assessing S. aureus killing (MOVIE_S31_FigS1_NP.mp4; MOVIE_S32_FigS1_Vancomycin.mp4). Movies S33–S36: Reactivation of fibrinolysis in S. aureus-induced sepsis favors IgG-mediated pathogen killing (MOVIE_S33_Fig7_PzP1.mp4; MOVIE_S34_Fig7_PzP1_IgG-.mp4; MOVIE_S35_Fig7_PzP1_uPA.mp4; MOVIE_S36_Fig7_PzP1_tPA.mp4).
