# Supplementary material for: A Multilayered Imaging and Microfluidics Approach for Evaluating the Effect of Fibrinolysis in Staphylococcus aureus Biofilm Formation
Source: Pathogens. 2023 Sep 6;12(9):1141. doi: 10.3390/pathogens12091141 (PMC10534389; doi:10.3390/pathogens12091141)
Supplement: Supplementary file 1 [file pathogens-12-01141-s001.zip › SupplementaryCaptions.pdf]

FIGURES AND CAPTIONS

Figure S1. Development of Imaging and microfluidics combined method to assess *S. aureus* adhesion, growth and biofilm formation. A, Microfluidic channels coated (30 min, r.t.) with *S. aureus* ($1.7x10^7$ CFU/ml) were used for experimental setup. Human serum (HS) used at 10 and 30% or human ACD-plasma (NP) at 1%, 3% and 10% in TSB were injected in the microchannels at 0.5 μl/min. **A,** *S. aureus* growth and biofilm formation are shown as relative GFP (upper) and PI (lower) MFI±SE as a function of time and normalized over the first analyzed time point. PI signal was considered superimposed to the GFP positive mask, as described in Material and Methods section. **B,** Circularity index (CI) of *S. aureus* colonies at different conditions. CI was calculated on the GFP mask. Insets, representative masks of *S. aureus* colonies for NP 1% and NP 10% at *t*=100 min and relative average CI. **C,** GFP and PI fluorescent images of **A** are shown at *t*=30, 100 and 180 min. Bar, 50 μm. **D,** GFP and PI relative MFI±SE over time in presence of 10% NP or vancomycin (50 μg/ml). NP, same data reported in panel **A**. **E,** Ratio between PI and GFP MFI±SE. **F,** GFP, PI images at *t*=180 min. Bar, 50 μm. Dashed inset, refer to areas referred to single colony detail showed on the right; Bar 10 μm. **A, B, C, D, E, F** refer to same series of experiments performed and each point is average of 3-8 ROIs from 3 pooled independent experiments using 10% NP (one experiment used also in Fig.3), whereas results from use of 1% and 3% NP, 10% and 30% HS were obtained in 1 independent experiment. $^{**}P<0.007$, $^{*}P<0.03$ GFP rMFI, PI rMFI, PI/GFP MFI ratio at *t*=180 min, CI: *t*> 100 min.

Figure S2. Irrelevance of host coagulation elements in *S. aureus* biofilm formation. Microfluidic channels coated with *S. aureus* ($1.7x10^7$ CFU/ml). 10% of NP, FVII-($FVII^-$) and FX-(FX^-)-depleted ACD-plasma were used. **A,** *S. aureus* growth (GFP rMFI, Mean±SE) and biofilm formation (PI rMFI, Mean±SE) are shown. 3-6 ROIs from a single experiment (out of 2 with similar results) were analyzed.

Figure S3. Detection of anti-*S. aureus* IgGs in plasma from septicemic patients. An indirect ELISA was used as described in Material and Methods. ACD-plasma of *S. aureus* -induced septicemic patients (PzP 1 and PzP 2) and NP were incubated at different dilutions on *S. aureus* lysate (1:100 of lysate from $1x10^8$ CFU/ml of *S. aureus*) in 96 well-plates. Results are reported as Mean O.D. (A_{405nm}) of triplicates.

Table S1 List of abbreviations.

Movies S1-S6: Role of fibrinogen in *S. aureus* adhesion. *[MOVIE_S1_Fig1_FG.mp4; MOV-IE_S2_Fig1_FN.mp4; MOVIE_S3_Fig1_HA.mp4; MOVIE_S4_Fig1_TypeIV_Col.mp4; MOV-IE_S5_Fig1_TypeI_Col.mp4; MOVIE_S6_Fig1_Ctrl.mp4]*

Movies S7-S11: Development of Imaging and microfluidics combined method to assess *S. aureus* adhesion, growth and biofilm formation. *[MOVIE_S7_FigS1_HS10%.mp4; MOVIE_S8_FigS1_HS30%.mp4; MOV-IE_S9_FigS1_NP10%.mp4; MOVIE_S10_FigS1_NP3%.mp4; MOVIE_S11_FigS1_NP1%.mp4]*

Movies S12-S16: Role of fibrinogen in assembly and formation of *S. aureus* biofilm. *[MOV-IE_S12_Fig2_NP.mp4; MOVIE_S13_Fig2_FG-.mp4; MOVIE_S14_Fig2_FG- +FG.mp4; MOV-IE_S15_Fig2_TSB.mp4; MOVIE_S16_Fig2_TSB+FG.mp4]*

Movies S17-S19: Plasminogen does not affect *S. aureus* biofilm formation. *[MOVIE_S17_Fig3_NP.mp4; MOVIE_S18_Fig3_FG-.mp4; MOVIE_S19_Fig3_PLG-.mp4]*

Movies S20-S22: Role of fibrinogen in adhesion, assembly and formation of biofilm by *S. aureus* in flow. *[MOVIE_S20_Fig4_NP.mp4; MOV-IE_S21_Fig4_FG-.mp4; MOVIE_S22_Fig4_FG- +FG.mp4]*

Movies S23-S26: Triggering fibrinolysis interferes in the formation of *S. aureus* biofilm. *[MOVIE_S23_Fig5_NP.mp4; MOV-IE_S24_Fig5_FG-.mp4; MOVIE_S25_Fig5_upa.mp4; MOVIE_S26_Fig5_tPA.mp4]*

Movies S27-S30: Triggering fibrinolysis interferes in initial phase leading to biofilm formation by *S. aureus* in flow. *[MOVIE_S27_Fig6_NP.mp4; MOVIE_S28_Fig6_FG-.mp4; MOVIE_S29_Fig6_upa.mp4; MOVIE_S30_Fig6_tPA.mp4]*

Movies S31-S32: Development of Imaging and microfluidics combined method to assess *S. aureus* killing. *[MOVIE_S31_FigS1_NP.mp4; MOV-IE_S32_FigS1_Vancomycin.mp4]*

Movies S33-S36: Reactivation of fibrinolysis in *S. aureus*-induced sepsis favors IgG-mediated pathogen killing. *[MOVIE_S33_Fig7_PzP1.mp4; MOV-IE_S34_Fig7_PzP1_IgG-.mp4; MOVIE_S35_Fig7_PzP1_uPA.mp4; MOV-IE_S36_Fig7_PzP1_tPA.mp4]*
