# Supplementary material for: A Multilayered Imaging and Microfluidics Approach for Evaluating the Effect of Fibrinolysis in Staphylococcus aureus Biofilm Formation
Source: Pathogens. 2023 Sep 6;12(9):1141. doi: 10.3390/pathogens12091141 (PMC10534389; doi:10.3390/pathogens12091141)
Supplement: Supplementary file 1 [file pathogens-12-01141-s001.zip › TabS1_List_of_abbreviations.pdf]

pList of abbreviations	
ACD	acid-citrate-dextrose
ECM	extracellular matrix
eDNA	extracellular DNA
HRP	horseradish peroxidase
PDMS	polydimethylsiloxane
TSB	tryptic soy broth
NP	normal plasma
HS	human serum
FG	fibrinogen
FG^-	fibrinogen-depleted plasma
FN	fibronectin
FVII	factor VII
$FVII^-$	factor VII-depleted plasma
FX	factor X
FX^-	factor X-depleted plasma
HA	hyaluronic acid
IgG	immunoglobulin
IgG^-	IgG-depleted plasma
PLG	plasminogen
PLG^-	plasminogen-depleted plasma
PzP	plasma from patient
uPA	urokinase plasminogen activator
tPA	tissue-type plasminogen activator
BF	bright field
GFP	green fluorescent protein
PI	propidium iodide
ROI	region of interest
MFI	median fluorescence intensity
rMFI	relative MFI
CI	circularity index
