# Supplementary figures and images for: A Multilayered Imaging and Microfluidics Approach for Evaluating the Effect of Fibrinolysis in Staphylococcus aureus Biofilm Formation
Source: Pathogens. 2023 Sep 6;12(9):1141. doi: 10.3390/pathogens12091141 (PMC10534389; doi:10.3390/pathogens12091141)

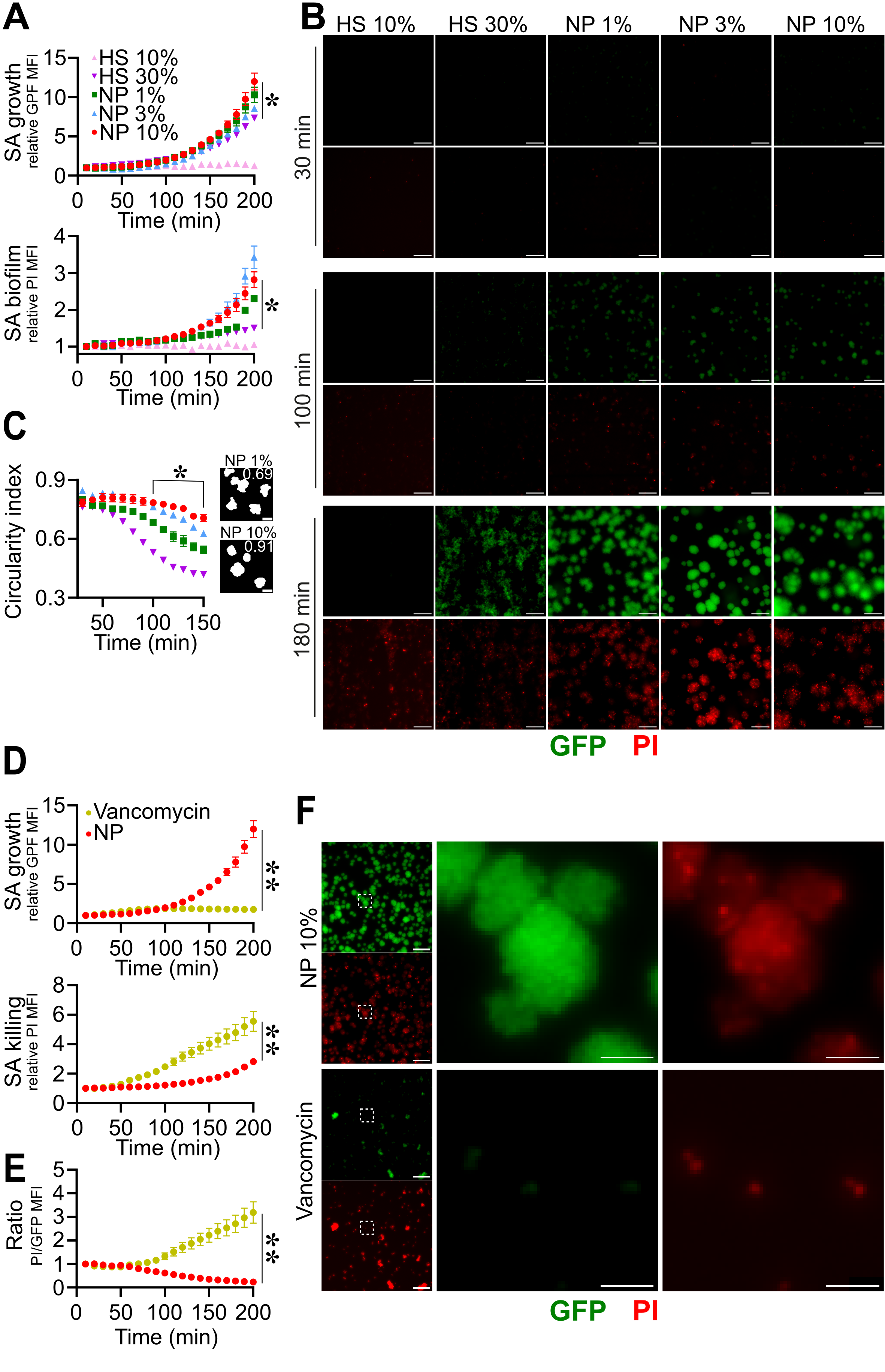

Supplement: Supplementary file 1 [file pathogens-12-01141-s001.zip › FigureS1.tiff]

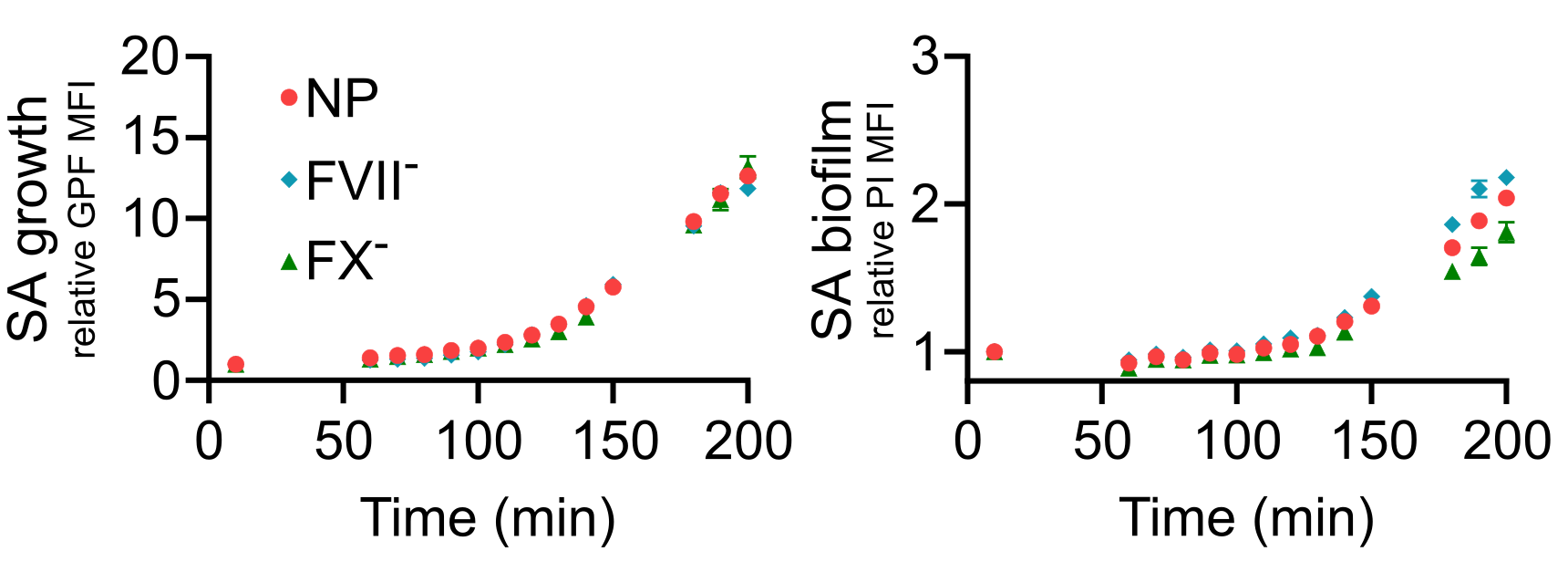

Supplement: Supplementary file 1 [file pathogens-12-01141-s001.zip › FigureS2.tiff]

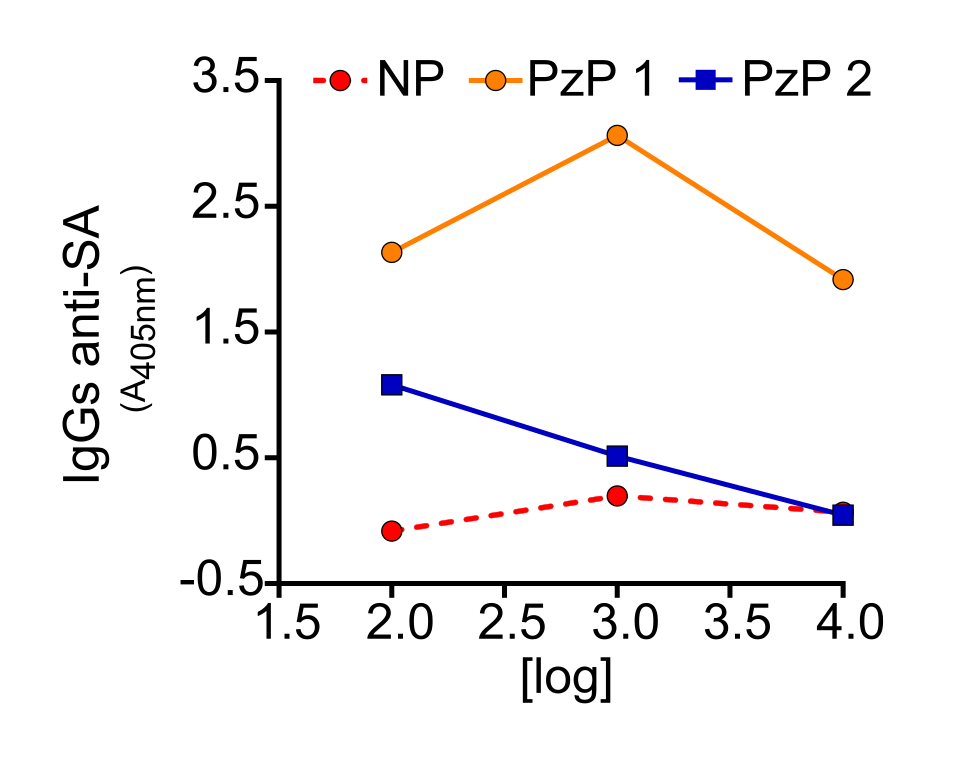

Supplement: Supplementary file 1 [file pathogens-12-01141-s001.zip › FigureS3.tiff]
